# Subarachnoid hemorrhage in the emergency department

**DOI:** 10.1186/s12245-021-00353-w

**Published:** 2021-05-12

**Authors:** Sima Patel, Amay Parikh, Okorie Nduka Okorie

**Affiliations:** Division of Neurocritical Care, Department of Critical Care Medicine, AdventHealth, 601 E Rollins St, Orlando, FL 32803 USA

**Keywords:** Subarachnoid hemorrhage, Emergency department, Neurologic injury, Hunt and Hess, Modified Fischer, Aneurysmal subarachnoid hemorrhage, Non-aneurysmal subarachnoid hemorrhages

## Abstract

**Background:**

Subarachnoid hemorrhage accounts for more than 30,000 cases of stroke annually in North America and encompasses a 4.4% mortality rate. Since a vast number of subarachnoid hemorrhage cases present in a younger population and can range from benign to severe, an accurate diagnosis is imperative to avoid premature morbidity and mortality. Here, we present a straightforward approach to evaluating, risk stratifying, and managing subarachnoid hemorrhages in the emergency department for the emergency medicine physician.

**Discussion:**

The diversities of symptom presentation should be considered before proceeding with diagnostic modalities for subarachnoid hemorrhage. Once a subarachnoid hemorrhage is suspected, a computed tomography of the head with the assistance of the Ottawa subarachnoid hemorrhage rule should be utilized as an initial diagnostic measure. If further investigation is needed, a CT angiography of the head or a lumbar puncture can be considered keeping risks and limitations in mind. Initiating timely treatment is essential following diagnosis to help mitigate future complications. Risk tools can be used to assess the complications for which the patient is at greatest.

**Conclusion:**

Subarachnoid hemorrhages are frequently misdiagnosed; therefore, we believe it is imperative to address the diagnosis and initiation of early management in the emergency medicine department to minimize poor outcomes in the future.

## Background

Headaches account for 2% of all emergency department (ED) visits, with subarachnoid hemorrhage (SAH) being one of the most dangerous diagnoses associated with mortality ranging from 8 to 65% [1–3]. Headache is one of the most common chief complaints in the ED. The diagnostic modalities for workup vary, and the etiologic possibilities are vast (Table 1).

**Table 1:** Differential diagnoses for sudden onset headache [2, 4]

• Subarachnoid hemorrhage (aneurysmal, arteriovenous malformation, traumatic, perimesencephalic, unknown etiology) • Meningitis/encephalitis • Temporal arteritis/complicated migraine • Cluster headache • Reversible cerebral vasoconstriction syndrome • Acute narrow-angle closure glaucoma • Hypertensive emergencies • Acute strokes: hemorrhagic or ischemic • Carbon monoxide poisoning • Idiopathic intracranial hypertension (pseudotumor cerebri) • Spontaneous intracranial hypotension • Cerebral venous and dural sinus thrombosis • Mass lesions • Cervico-cranial artery dissections • Pituitary apoplexy	

SAH typically presents with a sudden onset, severe headache and with patients textually describing it as “the worse headache of [their] life [4].” Studies report the range of total misdiagnosis of SAH from 12 to 51% [4]. One percent of all ED headaches are secondary to SAH; however, approximately 5.4% of patients diagnosed with SAH were initially misdiagnosed in the ED [4–6]. The decision to investigate a patient with neurologic deficits is unequivocal. It is challenging to determine if an alert and neurologically intact patient requires a more detailed initial workup. An emergency medicine physician must decide when to proceed with neuroimaging or a lumbar puncture when a history and physical are insufficient.

## Methods

### Data sources and search

We performed a PubMed search for subarachnoid hemorrhage articles published, using the following search terms: subarachnoid hemorrhage, subarachnoid hemorrhage in the emergency department, traumatic brain injury in the emergency department, emergency medicine brain injury, management of neurologic injury, management of SAH in the ED, and obstacles in diagnosing SAH.

### Study selection

Prospective data, observational data, cross sectional data, systemic reviews, meta-analysis, and guidelines on emergency medicine evaluation were included. Eighty papers were analyzed and those excluded focused on long-term management or treatments for subarachnoid hemorrhages that were outside the scope of emergency medicine. Cases only with SAH were cited. Eligible articles were published in English.

## Discussion

### Symptom considerations

Clinically, SAHs are classified as aneurysmal subarachnoid hemorrhage (aSAH) and non-aneurysmal subarachnoid hemorrhages (NASAH). Diagnosis is often missed due to the various clinical manifestations and inconsistencies in individual findings. Differential diagnosis of SAH should always be considered during the workup. There are several etiologies of non-traumatic SAH including perimesencephalic SAH, intracranial arterial dissection, pituitary apoplexy, mycotic aneurysms, reversible cerebral vasoconstriction syndrome (RCVS), cerebral venous sinus thrombosis, moyamoya, vasculitis, and even cocaine use.

Predictors of SAH based on exam and history can be found in Table 2 [5]. Patients can present with an acute, sudden onset headache with neck pain and stiffness (likelihood ratio of 4.1 and 6.6), altered consciousness, cranial nerve palsy, hemiparesis, cerebellar signs, papilledema, or even retinal hemorrhage [7, 8]. A headache will be the primary and sometimes the sole symptom in 70% of patients, and only 50% will present with the textbook “thunderclap” headache [8]. It is often difficult to assess which patients require a more extensive workup based only on the quality of a headache, especially given thunderclap headache has not been well described in the literature. The classic thunderclap headache is defined in several literature pieces as a sudden onset, severe, or rapidly escalating headache and the worst headache of the patient’s life [7, 9]. However, it is unclear if the term refers to each quality or the combination of all [7, 9]. Many patients will have a persistent headache for several days, and very few will have a resolution within hours [8]. No single portion of a history has an increased likelihood ratio for SAH identification [5]. Since consideration for further workup can pose a challenge, recent studies have suggested methods to make the process effortless.

**Table 2:** Physical exam and history predictors of SAH

Finding	Positive LR	Negative LR
Exam neck stiffness	6.59	0.78
Subjective neck stiffness	4.12	0.73
Lethargy	2.19	0.74
Vomiting	1.92	0.52
Similar headache in past	1.90	0.90
Onset of headache 1–5 min	1.79	0.88
“Worst headache of life”	1.25	0.24
Nausea	1.15	0.74
Onset of headache < 1 h	1.13	0.06
Photophobia	1.07	1.05
Diplopia	0.96	1.00
Onset of headache < 1 min	0.91	1.11
Family history of cerebral aneurysm	0.22	1.07
Absence of “worst headache of life”	--	0.36

*LR* Likelihood ratio

### Diagnosis

#### Computed tomography

**Fig. 1:**
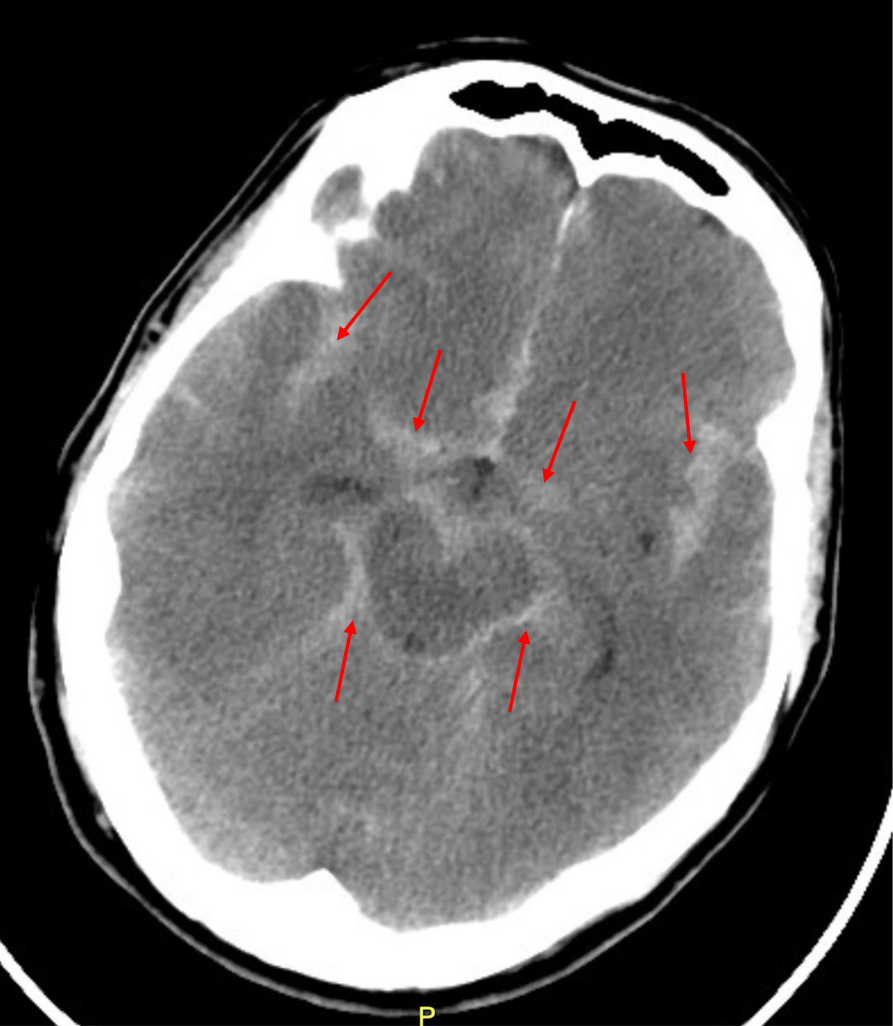
Subarachnoid hemorrhage on CT head

When SAH is suspected, computed tomography of the head (CTH) is the initial diagnostic measure [9] (Fig. 1). At the onset of bleeding, a CTH will be able to visualize blood more easily; it is difficult to appreciate blood on a CT after red blood cells begin to degrade [9]. The American Stroke Association suggests the sensitivity of a CTH within the first 3 days of insult remains approximately 100% [10]. The rate of negative CTHs increases over the next 3–7 days, necessitating a lumbar puncture [10]. The American College of Emergency Physicians’ recommendation on acute headaches is to utilize the Ottawa Subarachnoid Hemorrhage Rule to rule out SAH as it is highly sensitive for those with a normal neurologic exam with peak headache within 1 h of onset (level B recommendation) (Table 3) [11–13]. Unfortunately, it has a low specificity to rule in SAH for the same patient population [13]. Further observations recommend performing a CTH within 6 h of onset as the sensitivity to rule out SAH is > 99% (level B recommendation) [13, 14]. Many studies have noted cases where head CTs that were initially read negative for SAH were later read as positive for SAH. Therefore, it is imperative an experienced radiologist, perhaps a neuroradiologist, be assigned for adequate evaluation [15].

**Table 3:** Ottawa Subarachnoid Hemorrhage rule [11]

For alert patients > 15 years of age with new severe non-traumatic headache reaching maximum intensity within 1 h	
Not for patients with new neurologic deficits, previous aneurysms, SAH, brain tumors, or history of similar headaches (≥ 3 episodes over ≥ 6 months)	
Investigate further if ≥ 1 finding is present	
1. Symptoms of neck pain or stiffness	
2. Age ≥ 40 years old	
3. Witnessed loss of consciousness	
4. Onset during exertion	
5. Thunderclap headache (peak intensity immediately)	
6. Limited neck flexion on exam	

Perry and colleagues created the Ottawa SAH rule, which has almost perfect sensitivity for SAH [11, 16]. Moreover, the team determined that the 6-h CT rule [95.5% (95% CI, 89.8–98.5)] and the Ottawa SAH rule [100% (95% CI, 98.1%–100%)] are extremely sensitive, and both can be used when SAH is considered in patients presenting with headache [11]. Their study showed that implementing both rules decreased the need for testing and hospital admissions (*p* < 0.0001 and *p* < 0.011, respectively) [11]. Wu et al. and Bellolio et al. drew similar conclusions regarding sensitivity [17, 18]. Furthermore, lumbar puncture (LP) or CT angiography use was decreased as a result of the utilization of the 6-h CT rule and the Ottawa rule (*p* < 0.0001) [11].

Given the sensitive nature of providing this diagnosis, local or institutional limitations and radiologic factors should be considered when applying the 6-h rule. Many studies noted that facilities should implement a third generation or higher CT scanner for evaluation, an attending level radiologist with neuroradiology experience should be assigned for interpretation (as many trainees and nonexperts increase risk of error), CT images should be cut less than or equal to 5-mm slices and hematocrit should be greater than 30 [15]. If these criteria are met, then many consider a negative CTH to be a “rule-out” study [9].

#### Lumbar puncture

Classically, if history and symptoms contribute to increased clinical suspicion for SAH after a negative non-contrast CTH, a LP should be performed [19]. The proper procedure includes collecting at least four tubes of cerebrospinal fluid (CSF) for an adequate sample [20]. The American College of Emergency Physicians shares a level C recommendation to obtain an LP or a CT angiography of the brain to investigate patients at high risk for SAH in the setting of a negative CTH [13]. A lumbar puncture will reveal xanthochromia, indicating heme metabolism, in the cerebrospinal fluid (CSF), diagnostic of SAH [20, 21]. Given the timing of red blood cell breakdown to detect xanthochromia, literature suggests a LP can be performed up to 12 h from symptom onset [9]. Should the last tube confirm less than five red blood cells (RBCs), then SAH is excluded [20]. Conversely, should the last tube detect greater than 2000 × 10^6^ RBCs/liter (irrespective of a traumatic LP), then a CT angiography should be considered to assess for aneurysm [9]. Recent studies challenge LPs’ value in this setting, given the improvement in third-generation CT scanners and increased utility of CT angiographies [22].

It is extremely important to consider the limitations or even risks of a LP prior to performing it. It can be difficult to execute the procedure for several reasons, including the patient’s body habitus, previous history of spinal procedures, previous history of LPs in the past, and the fact that it is time-consuming in some cases [19]. Patients with distressing or painful past experiences can lead to a refusal, and complications such as epidural hematomas or post LP headache can lead to additional invasive procedures [19, 21]. Traumatic LPs, occurring in 10% of all ED LPs, can often obscure results leading to a misdiagnosis or requiring the physician to discard the results altogether [19, 20].

#### Additional considerations

Given the length of time patients spend in the ED prior to admission, ancillary testing beyond a non-contrast CTH and LP may not be feasible. Notably, many studies have considered replacing an LP for a CT angiography (CTA) of the brain in SAH diagnosis [23]. Since the CTA is far less risky than an invasive and painful LP, the admission team should seriously evaluate whether a LP is truly necessary. The American Heart Association/American Stroke Association states a CTA could be considered in the workup for suspected aneurysmal SAH (class IIb, level C recommendation) [10]. A CTA is beneficial because it can diagnose other causes of acute headache such as venous thrombosis, stroke, or arteriovenous malformation [23]. A CTA has been shown to be 100% specific and 98% sensitive for aneurysms greater than 3 mm [24]. Diagnosis and intervention of aneurysms have been shown to decrease patient morbidity, making it a worthy consideration in the ED setting [19]. A CTA’s limitations include incidental aneurysm diagnosis leading to unwarranted interventions, contrast exposure, and nephropathy risk [23, 25]. For these reasons, current ED guidelines do not recommend CTA as a diagnostic modality for SAH [25].

### Classification

Once the diagnosis of SAH hemorrhage is made, it is important to classify and grade the patient’s risk to guide the urgency of further management. In 1988, a grading system proposed by the World Federation of Neurologic Surgeons was originally intended to predict patient outcomes by using the Glasgow Coma Scale (GCS) and the absence or presence of major motor deficits (Table 4) [26]. A higher grade was associated with a worse outcome; however, more superior grading systems have been developed over the years [26].

**Table 4:** World Federation of Neurologic Surgeons (WFNS) Scale [26]

Grade	Glasgow Coma Scale	Motor deficit
1	15	Absent
2	13–14	Absent
3	13–14	Present
4	7–12	Present or absent
5	3–6	Present or absent

The Hunt and Hess classification was established in 1968 to estimate mortality based on a specific list of criteria [27, 28]. The classifications range from one to five, and each is associated with a mortality percentage (Table 5).

**Table 5:** Hunt and Hess Classification [27, 28]

Grades	Description	Mortality
1	Asymptomatic or minimal headache and slight nuchal rigidity	3%
2	Moderate or severe headache, nuchal rigidity, no neurologic deficit other than cranial nerve palsy	3%
3	Drowsiness, confusion, or mild focal deficit	9%
4	Stupor, moderate to severe hemiparesis, possibly early decerebrate rigidity, and vegetative disturbance	24%
5	Deep coma, decerebrate posturing, moribund appearance	71%

The modified Fisher scale incorporates radiographic findings based on the initial CTH to predict the incidence of vasospasm. The scale was derived from the original Fisher grading scale to account for the thickness of the hemorrhage and any associated intraventricular hemorrhage (IVH). The grading system is from zero to four, with a high score indicating an elevated incidence of vasospasm (Table 6) [27, 29]. Taking both scores into account after a prompt diagnosis will allow for timely and effective treatment initiation to avoid future complications.

**Table 6:** Modified Fisher Scale [29]

Grade	Criteria on CT head	Incidence of Symptomatic Vasospasm
0	No SAH; no IVH	0%
1	Focal or diffuse, thin SAH; no IVH	24%
2	Thin, focal, or diffuse SAH; IVH	33%
3	Thick, focal, or diffuse SAH; no IVH	33%
4	Thick, focal, or diffuse SAH; IVH	40%

*CT* Computed tomography, *SAH* Subarachnoid hemorrhage, *IVH* Intraventricular hemorrhage

### Management

#### Medical management

With the diagnosis of SAH comes the risk of early complications. The 1-year mortality for untreated SAH is nearly 65%, which is reduced to 18% with the appropriate diagnosis and initiation of treatment [9]. If left untreated, the 24-h mortality is 25%, making early diagnosis imperative [30]. Although several complications are associated with various SAH types (Table 7), here, we focus on the most discussed and well known [31].

**Table 7:** Medical complications of SAH

• Rebleeding • Vasospasm • Hyponatremia • Cerebral salt wasting • Seizures • Hydrocephalus • Herniation • Coma • Cardiogenic shock • Neurogenic stress cardiomyopathy	

Rebleeding should be considered the first and most dire complication of SAH in the ED setting. More than 40% of patients with SAH in the ED have a systolic blood pressure (SBP) ≥ 185 which will increase the risk of rebleeding as a result of a ruptured aneurysmal SAH [32]. The risk of rebleeding is the highest in the first 2–12 h of insult, with rates ranging from 4 to 13.6% in the first 24 h [10]. Rebleeding is associated with poor prognosis, increased mortality, and poor neurologic recovery in survivors [10]. Although there is no direct relationship between ED-reported SBP and rate of rebleeding, a meta-analysis reported patients with an SBP > 160 mmHg in the ED were more likely to suffer from early rebleeding than those with an SBP > 140 mmHg [32]. Therefore, prompt BP reduction and management are suggested, especially in patients with suspected unsecured aneurysms. The current recommendation as stated by the Neurocritical Care Society states to reduce the systolic blood pressure to less than 160 or mean arterial pressure less than 110 for an unsecured aneurysm [33]. It is a class I, level B recommendation by stroke guidelines for blood pressure control in patients with SAH and unsecured aneurysm with a titratable agent (i.e., nicardipine, clevidipine, labetalol) to balance the risk of hypertension-related bleeding and risk of stroke with sudden hypotension, and for maintenance of cerebral perfusion pressure [10].

One of the most recognized complications of aSAH is vasospasm. Even with adequate medical management, vasospasm increases the risk of ischemic injury, doubling the mortality risk in SAH [34]. Although many clinicians rush to initiate a calcium channel blocker to reduce the risk of vasospasm, the risk is truly the highest 7–10 days after the initial hemorrhage [10]. This risk can continue for up to 21 days after admission making vasospasm a longer-term consideration.

Additional medical management strategies include addressing pain and nausea to avoid valsalva effect and to concomitantly provide blood pressure control. Use of intravenous medications with short half-lives is recommended (i.e., fentanyl) [9]. Antiepileptics should be considered in patients diagnosed with SAH. Approximately 20% of patients with SAH suffer a seizure prior to arrival and another 5–10% experience a seizure after admission [33]. Currently, there are no direct recommendations on specific anti-epileptic utilization for prophylaxis in SAH. Both American Heart Association (AHA) and Neurocritical Care Society (NCS) recommend the use of a short course of prophylactic anti-epileptic in the immediate post-hemorrhage period.

#### Coagulopathy reversal

In the age of novel and direct oral anticoagulation, the reversal of these agents plays a major role in treatment of SAHs in the ED and should be achieved as soon as possible. Table 8 provides a basic reference to mainstay anticoagulation reversal agents and mechanisms of action [35]. Vitamin K antagonists can additionally be reversed with phytonadione (vitamin K) or fresh frozen plasma (FFP). However, prothrombin complex (PCC) is preferred given it does not require blood bank typing, it has a rapid onset, and it has a low volume of infusion [9]. NCS recommends the use of platelet transfusion in those taking aspirin or adenosine diphosphate inhibitors who will undergo neurosurgical intervention [36]. Regardless of anticoagulation agent, we encourage institutional protocols be implemented for effective decision making and rapid administration of reversal agent in the setting of SAH.

**Table 8:** Anticoagulation reversal agents

Target agent	Reversal agent	Reversal agent mechanism of action	Dosing
Vitamin K antagonists	3 factor or 4 factor prothrombin complex (PCC)	Replacing factors II, IX, X, and VII (4-factor PCC); protein C, S, and Z in other products	25–50 U/kg intravenous
Factor Xa inhibitors, heparin, low-molecular weight heparin (LMWH), and fondaparinux	Andexanet alfa	Recombinant variant of human factor Xa that competes with native factor Xa for binding of rivaroxaban, apixaban, and edoxaban, and the heparin-, low-molecular weight heparin-, and fondaparinux-antithrombin complex	400 mg intravenous bolus followed by intravenous infusion of 480 mg over 2 h for reversal of apixaban or rivaroxaban if > 7 h previously 800 mg followed by an infusion of 960 mg over 2 h for those taking rivaroxaban or rivaroxaban if ≤ 7 h previously or edoxaban
Dabigatran	Idarucizumab	Noncompetitive, specific, and direct binding of dabigatran	5 g intravenous bolus
Antiplatelets	Platelets or desmopressin	Desmopressin increases release of von Willebrand factor in platelets thus increasing factor VIII availability for clotting	0.4 mcg/kg Uremia, 0.3 mcg/kg

*g* Grams, *mcg* Micrograms, *kg* Kilogram, *mg* Milligram, *h* Hour, *U* Units

Studies date back to 1979 if utilization of tranexamic acid (TXA) is beneficial in SAH. A double-blind study by Kaste and colleagues, determined there were no differences in rebleeding, morbidity, or mortality with the utilization of tranexamic acid versus placebo in SAH patients [37]. Over additional years, many began to ask if the use of TXA changed patient outcomes in the setting of SAH. Roos et al. concluded that the use of TXA with standard treatment did not improve patient outcomes [38]. In 2021, the ULTRA trial also concluded that the use of tranexamic acid did not show improved clinical outcomes defined by the modified Rankin score (mRS) at 6 months [39]. The Neurocritical Care Society does endorse that TXA and aminocaproic acid can reduce aneurysmal re-rupture; however, they have also been shown to increase risk of deep venous thrombosis, pulmonary embolus, and ischemic stroke if used for prolonged periods of time [33]. Although the use of TXA is not standard of care, it can be considered a strategy dependent on the case and local availability.

#### Surgical management

**Fig. 2:**
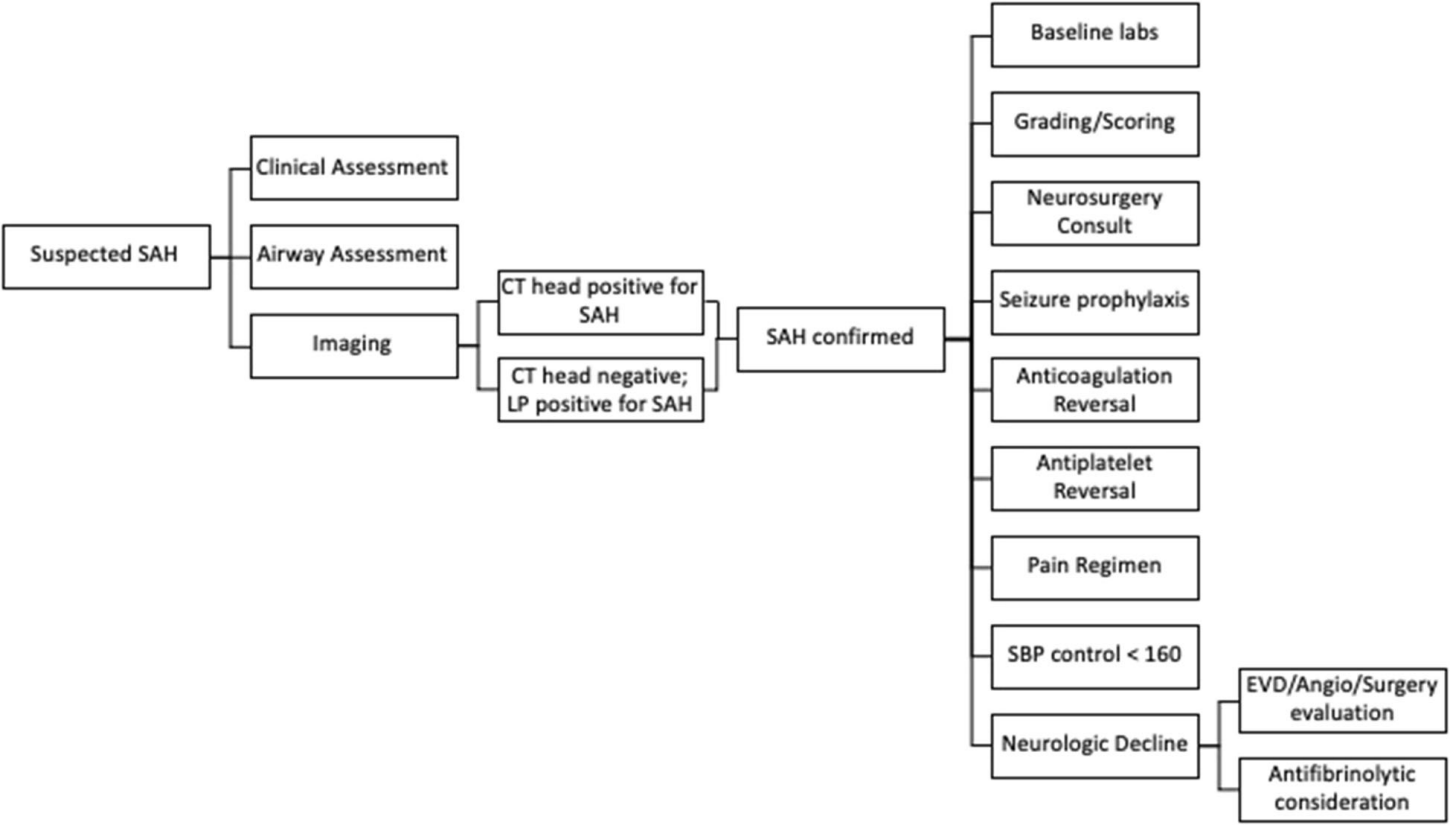
Subarachnoid hemorrhage management algorithm

Prior to definitive surgical management, it is important to note if reports of hydrocephalus or intraventricular hemorrhage are found on imaging. This should prompt an emergent neurosurgical evaluation for possible external ventricular drain (EVD). A management algorithm derived from the emergency neurologic life support (ENLS) can be found in Fig. 2 [33].

Endovascular coiling and open surgical clipping are two primary approaches to aSAH [31]. The choice of intervention is determined by several variables, including the patient’s complications and aneurysm anatomy, and the availability of surgical expertise [10]. The International SAH Trial (ISAT) compared each intervention and determined that endovascular coiling showed patients were free of disability at 1 year after the intervention [10]. 23.5% of patients in the endovascular coiling group were disabled at 1 year compared to 30.9% of the surgical clipping group (*p* = 0.0001) [40]. ISAT showed the long-term risks of further bleeding after 1 year were low with either therapy (2 per 1276 in the endovascular group and 0 per 1081 in the surgical group) [40]. Given this finding, it is important to consider whether a patient would benefit from transfer to a high-volume medical center capable of advanced procedures.

## Conclusion

Subarachnoid hemorrhages carry a high risk of morbidity and mortality, requiring emergency medicine physicians to evaluate patients suspicious for the diagnosis cautiously [33]. It is important to consider the limitations of diagnostic modalities and early implementation of grading/scoring systems even in an atypical presentation. Given the SAH complications, making a timely diagnosis, initiating management in the ED, and employing suitable consultations/admission for possible early intervention is crucial for care.

## Data Availability

Not applicable

## Abbreviations

SAH: Subarachnoid hemorrhage
ED: Emergency department
aSAH: Aneurysmal subarachnoid hemorrhage
NASAH: Non-aneurysmal subarachnoid hemorrhages
CTH: Computed tomography of the head
LP: Lumbar puncture
CTA: CT angiography
GCS: Glasgow coma scale
SBP: Systolic blood pressure
ISAT: International SAH trial
WFNS: World federation of neurologic surgeons
TXA: Tranexamic acid
PCC: Prothrombin complex
FFP: Fresh frozen plasma
RCVS: Reversible cerebral vasoconstriction syndrome
ENLS: Emergency neurologic life support
EVD: External ventricular drain
